# The Feasibility of Outdoor Psychology Sessions in an Adult Mental Health Inpatient Rehabilitation Unit: Service User and Psychologist Perspectives

**DOI:** 10.3389/fpsyg.2021.769590

**Published:** 2021-12-23

**Authors:** Gail James, Katherine Kidd, Sam J. Cooley, Kelly Fenton

**Affiliations:** ^1^Department of Neuroscience, Psychology and Behaviour, University of Leicester, Leicester, United Kingdom; ^2^Department of Clinical Psychology, Leicestershire Partnership NHS Trust, Leicester, United Kingdom

**Keywords:** outdoor therapy, clinical psychology, inpatient mental health services, COVID-19, nature exposure

## Abstract

Few studies have explored outdoor therapy when facilitated by clinical psychologists within an inpatient mental health service. In the present study, outdoor psychology sessions were introduced after service users (SUs) expressed a desire to return to face-to-face working during the COVID-19 pandemic. This study aimed to explore SUs’ and clinical psychologists’ perspectives on the feasibility of conducting outdoor therapy within the service. A mixed-method approach was underpinned by critical realist philosophy. Three psychologists maintained reflective diaries following outdoor therapy sessions with 16 SUs. A subsample of 14 SUs completed scales measuring therapeutic alliance and comfort during outdoor sessions. A subsample of eight SUs participated in semi-structured interviews. Data was analysed using descriptive statistics and thematic analysis. Quantitative and qualitative data demonstrated high SU satisfaction with therapeutic alliance and comfort outdoors. Six themes were identified: utilising a person-centred approach; the value of multi-disciplinary team support; enhancing therapeutic engagement; the benefits of time away from the ward; managing confidentiality; physical health and safety. This feasibility study demonstrated the introduction of outdoor psychology sessions within an inpatient mental health service to be a viable response to COVID-19. The findings suggest outdoor therapy can be an effective and safe mode of therapy, and can offset the challenges of indoor working, providing certain risk factors are considered and managed. The limitations of this study and implications for clinical practice are discussed. Further research is now required to support future integration into clinical practice.

## Introduction

According to the biophilia hypothesis, humans have an innate need for contact with nature to achieve satisfaction and meaning in multiple areas of their lives (Kellert and Wilson, 1994). Nature has been identified to have preventative and restorative benefits for a range of mental health needs (Bowler et al., 2010; Engemann et al., 2019, 2020). Various theories have attempted to explain these effects. The Attention Restoration Theory argues that executive-functioning and self-regulation can be improved from spending time in nature, as this allows fatigued directed attention mechanisms time to replenish (Kaplan, 1995; Kaplan and Berman, 2010). The Stress Reduction Theory (Ulrich et al., 1991) posits that unthreatening natural environments have a calming effect that supports the recovery from stress through eliciting positive feelings and reducing physiological arousal.

Nature-based interventions, such as horticultural therapy, gardening, and the Japanese practice of Shinrin-yoku (“forest bathing”), have received increased attention internationally and are being introduced as methods of treating ill mental health (Clatworthy et al., 2013; Cipriani et al., 2017; Kotera et al., 2020). Talking therapy in natural, outdoor spaces has also become increasingly popular. A recent meta-synthesis examined the experiences of practitioners and service users (SUs) who engaged in outdoor talking therapy (Cooley et al., 2020). The review identified various benefits for the therapeutic process, including an increased sense of freedom for emotional expression, mind-body holism, interconnectivity with nature, added mutuality between therapist and SU, and improved practitioner wellbeing (Cooley et al., 2020).

Cooley et al. (2020) identified a significant gap in the current outdoor therapy literature. Most research in the review was conducted in private or educational settings rather than in public health organisations, limiting access to outdoor therapy to those who can afford private therapy. Organisational barriers were cited as a likely reason for the lack of research into outdoor therapy in public healthcare services such as the National Health Service (NHS). The review called for case studies of public healthcare organisations who were introducing outdoor therapy to illuminate any barriers and facilitators to outdoor practice.

Adult inpatient mental health rehabilitation services in the United Kingdom aim to support individuals with complex and enduring mental health difficulties to develop the emotional, practical and social skills needed for successful community reintegration and independent living (Killaspy et al., 2005; Killaspy and Zis, 2013). The COVID-19 pandemic resulted in the government enforcing a number of safety measures, including physical distancing and reduced face-to-face contact (Public Health England, 2021). These restrictions impacted how inpatient services offer talking therapies and initially led to digital alternatives (e.g., telephone and video calling; British Psychological Society, 2020a). However, it has since been identified that digital therapy is not suitable for all SUs with long-term mental health difficulties (Miu et al., 2020). Therefore, additional alternatives to indoor therapy need to be offered when possible to promote engagement. The British Psychological Society (BPS) published guidance on how to effectively and safely conduct talking therapy outdoors (British Psychological Society, 2020b). This method of therapy reduces the risk of infection and aligns with inpatients’ desires for more opportunities to go outside (Molin et al., 2016).

The present study took place across two inpatient units, with 40 open ward beds and eight high dependency unit beds, within an NHS adult inpatient mental health rehabilitation service located in central England. Prior to the COVID-19 pandemic, talking therapy was predominantly held indoors, with occasional outdoor sessions based on patient need (e.g., graded exposure during Cognitive Behavioural Therapy). During the COVID-19 pandemic, the Clinical Psychology department initially moved to offering digital therapy. However, anecdotal evidence from SUs suggested many individuals found phone sessions were not as effective as face-to-face working. After identifying empirical evidence that highlighted the benefits of taking therapy outdoors, the team began to offer outdoor psychology sessions in line with the aforementioned BPS guidance. Given the lack of prior research in public healthcare settings and the novelty of offering more routine outdoor therapy within the present service, a feasibility study was conducted to explore the impact of this change in practice for both SUs and psychologists.

## Method

### Participants

Two clinical psychologists and one trainee clinical psychologist from the rehabilitation service participated in the study and conducted outdoor sessions with 16 SUs (see Table 1 for SU characteristics). SUs were excluded from participation if they did not have leave to access the outdoors with their psychologist due to clinical risk or the SU being too unwell. The inclusion criteria was any inpatient SU with appropriate leave, who was actively involved with psychology and consented to engage in outdoor psychology sessions. Of the 16 SUs, 14 completed at least one outcome measure and eight participated in semi-structured interviews. All participants provided informed written or verbal consent before participation in this study. Ethical approval was granted by the NHS Trust’s research and development group.

**TABLE 1:** Service user characteristics.

**Gender, *n* (%)**	
Men	10 (62.50)
Women	5 (31.25)
Transgender	1 (6.25)
**Age (years)**	
Range	24–64
Mean (SD)	43.13 (13.19)
**Ethnicity, *n* (%)**	
White British	12 (75.00)
Indian	2 (12.50)
Black British/African	1 (6.25)
Mixed race	1 (6.25)
**Ward type, *n* (%)**	
High dependency unit	2 (12.50)
Open ward	14 (87.50)
**Mental health act status**	
Section 3	8 (50.00)
Informal	8 (50.00)
**Primary psychiatric diagnosis, *n* (%)**	
Acute and transient psychotic disorder	1 (6.25)
Paranoid schizophrenia	7 (43.75)
Bipolar affective disorder	2 (12.50)
Schizoaffective disorder	2 (12.50)
Depression	1 (6.25)
Recurrent depressive disorder	1 (6.25)
Dissociative motor disorder	1 (6.25)
Emotionally unstable personality disorder	1 (6.25)
**Co-morbid diagnosis, *n* (%)**	
Childhood autism	1 (6.25)
Attention deficit hyperactivity disorder	1 (6.25)
Generalised anxiety disorder	2 (12.50)
Anxiety disorder unspecified	1 (6.25)
Recurrent depressive disorder	1 (6.25)
Depressive episode	1 (6.25)
Substance misuse	4 (25.00)
Adjustment disorder	2 (12.50)
Paranoid personality disorder	1 (6.25)

### Materials and Procedure

Service users consented to engage in outdoor therapy as part of their treatment as usual. Before the initial outdoor session, psychologists and SUs completed a shared “contract” outlining how to proceed with outdoor therapy, in line with BPS guidance (British Psychological Society, 2020b). Psychologists maintained a reflective diary following each initial contracting session and subsequent outdoor sessions.

Sessions were facilitated in a range of outdoor environments, depending on leave restrictions and SU preference. Some sessions included sitting or walking on hospital grounds. The hospital grounds included green areas with footpaths and benches, as well as grass areas to sit and walk on. The benches on hospital grounds were near the hospital buildings. Other sessions were facilitated in the local community, with SUs and psychologists walking to the local shops or public park.

Following each outdoor session, SUs were asked to complete a Session Rating Scale (SRS V.3.0; Miller et al., 2000). The SRS is a four-item visual scale designed to measure four aspects of therapeutic alliance (relationship, goals and topics, approach and method, overall). SUs rate each item by marking a line on a scale, which yields four scores between 0 and 10 (higher scores indicate higher satisfaction). An additional question was added to the scale to assess how comfortable/uncomfortable SUs felt outdoors (0 = *I felt very uncomfortable outside* to 10 = *I felt very comfortable outside*). Research has mainly investigated the reliability and validity of the SRS with outpatients, university students and couple/family client groups, with the SRS demonstrating moderate to high internal consistency (α ranged from 0.70 to 0.97), good test-retest reliability (*r* ranged from 0.54 to 0.70) and low to moderate concurrent validity (see Murphy et al., 2020 for full review).

After engaging in at least one outdoor psychology session, SUs were invited to participate in a semi-structured interview about their experience. Interviews lasted between 30 and 45 min, depending on the time needed to gain an understanding of a SU’s experience. Interviews were conducted by a psychologist that was not the SU’s regular psychologist. Interviews were held either face-to-face (*n* = 6) or over the phone (*n* = 2). With SUs’ consent, answers were written verbatim by the interviewer and were regularly reviewed with the SU during the interview to ensure the content correctly captured their experience.

The qualitative data was analysed using thematic analysis (Braun and Clarke, 2006), underpinned by critical realist ontology (Guba and Lincoln, 1994). The analysis was led by author GJ, with the other authors acting as ‘critical colleagues’ to support collaborative reflexivity (Braun and Clarke, 2019).

## Results

### Descriptive Statistics

Fourteen SUs completed the SRS for between one to six sessions per SU, resulting in 43 scales being completed in total. Table 2 displays descriptive statistics for each SRS item, including the additional item for outdoor comfort. Mean scores indicate the majority of SUs rated high scores for satisfaction with therapeutic alliance and comfort outdoors. Clinical records indicated that the SUs who reported lower scores for comfort outdoors were undergoing an exposure intervention to support high levels of anxiety toward outdoor environments.

**TABLE 2:** Descriptive statistics for SRS scores.

SRS item	Mean (standard deviation)	Range of scores
Relationship	8.97 (1.26)	4.7–10
Goals and topics	8.88 (1.23)	4.9–10
Approach and method	9.18 (1.00)	6.5–10
Overall	8.80 (1.50)	4.4–10
Comfort outdoors	8.13 (2.44)	0–10

### Qualitative Analysis

Six main themes were identified across the SU interviews and psychologists’ diaries (see Table 3).

**TABLE 3:** Themes.

Theme
Utilising a person-centred approach.
The value of multi-disciplinary team support.
Enhancing therapeutic engagement.
The benefits of time away from the ward.
Managing confidentiality.
Physical health and safety.

#### Utilising a Person-Centred Approach

Psychologists regularly adapted to SUs’ needs and reasons for engaging in outdoor therapy during the planning and facilitation. Due to the COVID-19 pandemic, restrictions initially led to phone appointments being offered, and the length of indoor sessions being limited to 30 min. Following introducing the option of 60-min outdoor sessions, SUs reported they “prefer to see my psychologist in person, rather than on the phone” (Service User [SU] 1) and were “keen to have outdoor therapy due to the longer appointment time” (Psychologist [P] 3). Although, this was noted to be a compromise for one SU who wanted to return to face-to-face working but did not have a desire to meet outdoors. For other SUs being outdoors and physically active was important to them and therefore outdoor working was “in line with their personal preference and values” (P3). Additionally, spending time outdoors was part of some SUs’ rehabilitation goals (e.g., “This person’s goals are to go out into the community more.” P1) and therefore was integral to their psychology intervention (e.g., graded exposure).

Decisions around whether outdoor sessions should include sitting or gentle walking (in the community or on hospital grounds) were informed by SUs’ risk assessments, formulations and preferences. For example, “knowing this person’s formulation, having sessions where I was required to push [their] wheelchair for longer distances…would be psychologically challenging for [them]. Therefore, I did not suggest walking sessions, just outside sat down sessions” (P1). The intended objectives of a session also contributed to the choice of activity (e.g., sitting on a bench to review a report).

The extent the natural environment was incorporated into sessions varied according to SU need. Some sessions used the natural environment as a backdrop, with observations about the surroundings sometimes spontaneously being commented on in conversation. On other occasions, the environment was actively used during the session to support SUs to manage their distress or engage in relaxation techniques as part of an intervention (e.g., “It relaxed my mind… we used what we could see, hear, touch around us” SU3).

Psychologists discussed reviewing how sessions were progressing and re-contracting if necessary. For example, “We agreed that if they… felt uncomfortable at all, they would let me know so we could review how to manage these situations” (P3). This highlighted how psychologists attempted to ensure a collaborative approach was maintained throughout the outdoor therapy process.

#### The Value of Multi-Disciplinary Team Support

Both psychologists and SUs highlighted the importance of continued multi-disciplinary team (MDT) working throughout the planning and facilitation of outdoor therapy. In an inpatient context, it is a legal requirement that SUs on a mental health section have the appropriate leave to engage in activities off the ward. Further, thorough risk assessments to ensure it is safe for SUs to engage in outdoor sessions must be conducted. This involves the collaboration of the MDT, including psychologists, consultant psychiatrists, nurses, and occupational therapists, and was reported by psychologists as crucial in the planning process.

Multi-disciplinary team members assisting SUs to go outdoors in between psychology sessions also built SUs’ familiarity with an outdoor setting and made the facilitation of outdoor therapy more feasible (e.g., “The fact that the person had tried doing this with other professionals also helped them to be willing to try it today” P1). Psychologists noted having “someone available to call [on the ward]” (P1) was reassuring and helpful in the event any risks arose during a session. One SU also shared “another member of staff from the ward came on the walk too because I was on escorted leave” (SU2). This highlights how the accessibility of colleagues during sessions also supported the safe facilitation of outdoor therapy.

#### Enhancing Therapeutic Engagement

Outdoor sessions promoted engagement in psychology through increased sense of freedom, self-regulation and mutuality in the therapeutic relationship. SUs reported psychology sessions in a clinic room as “too official” (SU5) and that having “nowhere to look” (SU2) heightened anxiety. The noise and disturbances on the ward were described as distracting, which made it difficult to engage in sessions.

In contrast, SUs reported having their psychology sessions outdoors helped them experience a sense of freedom and was “good for my anxiety” (SU3). The natural environment was repeatedly cited by SUs as supporting them to feel relaxed and safe, particularly being in the fresh air and “noticing the wildlife” (SU2). SUs reported “the ability to breathe easier” (SU8) outdoors, which helped them “think more clearly” (SU4) and made it easier to express themselves and talk to their psychologist.

Psychologists noted that several SUs seemed to find freedom of movement outside helpful to self-regulate during sessions. This was beneficial for SUs who felt restricted when having to sit during sessions and who found engaging in emotive conversations particularly challenging (e.g., “Whilst sitting they had been struggling to regulate their distress, but it appeared to become slightly easier when they started walking” P1).

In comparison to indoor sessions, SUs also described outdoor psychology sessions as being “easier to talk side on, less overwhelming, less eye-contact.” (SU1) and “I was able to vape, which gave me something to do and time to pause and gather my thoughts, rather than having an uncomfortable, awkward silence.” (SU2). This suggests SUs felt less pressure to comply with social expectations during outdoor sessions, which supported them to feel more comfortable in the company of their psychologist.

Several SUs described how being in an outdoor setting positively impacted their therapeutic relationship with their psychologist, including that it felt more “friendly and respectful” (SU5). Psychologists recognised that the more informal outdoor setting was “great in many ways because it reduces the barrier between “patient” and “staff” and helps input to feel more collaborative” (P1). However, the informal setting was also reported as a risk factor that might unintentionally give SUs the impression that the relationship was more friendship-based than therapeutic. Therefore, this was considered important to hold in mind and actively monitor during outdoor sessions.

#### The Benefits of Time Away From the Ward

Service users were often enthusiastic about having outdoor sessions and expressed enjoyment in having an opportunity to leave the ward. It was noted by SUs that “if you’re boxed into a place you can get claustrophobic” (SU6), but “once you’re out of the unit, it’s like a weight has been lifted temporarily… It’s nice not being in the pressure cooker of a hospital” (SU2). Psychologists also reflected how they felt more relaxed being away from the hospital and comfortable facilitating sessions outdoors (e.g., “[it] felt a little more relaxed than being in an office.” P2).

The inpatient ward were described as highly emotive and unpredictable environments, which is compounded by SUs complex and varying needs. One psychologist noted that leaving the ward may enable SUs to discuss incidents that occur on the ward more easily: “There had been a distressing incident on the ward the night before… I think being away from the ward helped when discussing this.” (P3). This, in turn, may provide more opportunity to process such events.

Outdoor sessions were also helpful in supporting SUs who found being outdoors anxiety provoking to begin to regulate their anxiety. Some SUs shared that having an opportunity to leave the ward with their psychologist contributed to building their confidence being outside and how this supported them toward discharge (e.g., “Some days you don’t feel like facing the outside, because of your anxiety, so going with a professional is a good way to break back into the community” SU2). One psychologist described noticing a difference in SUs’ tolerance of being outdoors: “We went further than the previous two sessions” (P1). Another SU reported having continued to go on regular walks as outdoor sessions had “re-sparked my love of nature and wanting to be outside” (SU1).

#### Managing Confidentiality

One of the challenges identified during outdoor sessions was maintaining SUs’ confidentiality due to the potential for other people to be present within the outdoor space. SUs had contrasting opinions around how to manage confidentiality during outdoor sessions. Some SUs reported experiencing worry about whether therapeutic conversations would be overheard and contracted with their psychologist that they would pause the conversation when others were in earshot. These concerns included SUs who required 2:1 escorted leave, which made creating a private therapy space outdoors even more challenging. For example, one SU said that having an additional staff member accompany them and their psychologist “made me a bit more self-aware, paranoia would kick in, I asked my psychologist ‘is he going to think any different of me?”’ (SU2); implying this arrangement was at times distressing and distracting for the SU. However, managing confidentiality can also be challenging during indoor sessions when SUs require 2:1 observations as members of the nursing team are also present during psychology sessions.

In contrast, other SUs advised they did not mind their conversations being overheard, therefore handing responsibility over to the psychologists to monitor appropriate levels of privacy (e.g., “I tried to pause a bit or be mindful of the language and questions I was asking when someone walked passed” P3). Psychologists’ phrasing of questions was also reported by SUs to help ease their worries about their confidentiality being maintained.

Stopping therapeutic conversations to maintain privacy was identified to have benefits and challenges. One psychologist stated “we engaged in problem-free talk which was a helpful opportunity to bring humour into the session and build rapport” (P3). Another psychologist reported the pauses gave a SU time to think carefully about their response to a question. The main challenges identified by psychologists were that pausing disrupted the flow of conversation and other people’s presence made sessions feel less private. Conversely, SUs expressed frustration that “people keep earwigging and wanting to hear what you are saying” (SU6) when having sessions on the ward. Therefore, outdoor sessions felt “a little more private than someone walking past the door” (SU8).

#### Physical Health and Safety

Having the opportunity to exercise was highlighted by SUs and psychologists as a physical health benefit to having sessions outdoors. A SU also shared they “felt a bit docile that morning because of my medication but being outdoors helped wake me up” (SU1). This suggests outdoor sessions can support SUs to manage medication side effects. However, when less physically active outdoor sessions needed to be facilitated, such as for SUs with less physical mobility, psychologists found there were limited seating areas on the hospital grounds that were comfortable and private, thus reducing the choice of locations. In some cases, sessions were finished indoors where appropriate seating was more readily available.

The weather also presented physical risks to outdoor working, such as slipping on wet and muddy ground and SUs not wearing skin protection in the sun. One psychologist expressed concern for their own wellbeing “as I have a physical health condition that deteriorates when I am cold” (P1). One SU shared “the pollen didn’t get to my nose and make me sneeze” (SU1). However, this raised a valid issue that those who have hay fever may experience more discomfort outdoors when the pollen count is higher. The need for appropriate equipment on the ward to maintain staff and SU safety in all types of weather was emphasised: “It made me think about the things we will need to be safe outside (e.g., sunscreen, hats, umbrella, coat, gloves etc.). We don’t always have these things in the workplace” (P2). At times, low temperatures, rain and inappropriate clothing led to outdoor sessions being moved indoors.

In line with COVID-19 physical distancing guidance at the time of the study, psychologists and SUs needed to maintain a two-metre physical distance from one another. This physical distance was problematic at times, such as on one occasion when a psychologist had to walk in the road due to narrow pathways. Busy traffic also presented another physical hazard, particularly when SUs’ and psychologists’ walking speed was different. This resulted in psychologists and SUs occasionally misjudging the time needed to safely cross a road (e.g., “I started to cross the road… and then realised that at the patient walking speed we wouldn’t have time to cross” P1).

## Discussion

Outdoor psychology sessions were introduced within an inpatient mental health rehabilitation service following anecdotal feedback from SUs that phone sessions were not as effective as meeting face-to-face during the COVID-19 pandemic. Given the lack of prior research in public healthcare settings that investigate the barriers and facilitators to outdoor practice, this study aimed to explore SUs’ and clinical psychologists’ perspectives on the feasibility of this change in practice as a response to COVID-19.

Qualitative feedback from SUs supported the SRS mean score ratings which suggested high satisfaction in both therapeutic alliance and comfort outdoors. However, some SUs did at times rate lower satisfaction levels. Lower levels of satisfaction were observed to usually be rated by SUs who were engaging in outdoor therapy as part of a Cognitive Behavioural Therapy exposure intervention. This suggests there is a strong clinical need for outdoor sessions to be considered even with SUs who may experience discomfort.

Service users’ enthusiasm for going outdoors and relief in having time away from the ward reflected previous research where inpatients expressed a desire for more opportunities to go outside (Molin et al., 2016). The experiences described by SUs when outdoors aligned with the theoretical understandings of how spending time in nature can support people’s wellbeing. SUs reported a sense of freedom and privacy outdoors, which lowered levels of distress and helped them relax during psychology sessions. This suggests SUs experienced emotional and physiological restoration as postulated by the Stress Reduction Theory (Ulrich et al., 1991). Psychologists also identified SUs found it easier to self-regulate in an outdoor environment, suggesting their cognitive functioning was supported by being outdoors, as theorised by the Attention Restoration Theory (Kaplan, 1995). These positive outcomes increased SUs’ level of comfort in talking to their psychologist, which could be challenging in a formal clinical room on the busy ward. This was further supported by SUs who rated higher satisfaction in therapeutic alliance on the SRS. These findings reflect previous research which identified indoor clinical settings can be intimidating and anxiety provoking for SUs (Jordan and Marshall, 2010), whilst outdoor sessions can promote emotional expression and mutuality in the therapeutic relationship (Cooley et al., 2020).

Due to the unpredictability of outdoor working, risks and practical issues were identified during outdoor sessions. These included challenges in maintaining confidentiality and relational boundaries, and keeping physically safe in different types of weather and environments. These risks were cited to occasionally disrupt outdoor sessions, but were not reported to be overly hindering and were usually manageable within sessions. Using a person-centred approach and working alongside the MDT was recognised as essential in supporting the feasibility of safe and effective outdoor sessions. Collaborative working allowed for potential risks and SU needs and preferences to be identified, managed and reviewed throughout the course of outdoor therapy. This way of working likely supported SUs to feel comfortable in an outdoor setting and experience satisfaction in the therapeutic alliance, as suggested by SUs’ SRS mean score ratings. Therefore, with regular risk assessments it is likely risks can be effectively minimised, as outlined in the BPS guidance (British Psychological Society, 2020b).

Findings specific to the client group were also identified. Outdoor sessions were cited to support SUs to manage side effects of medication and build confidence being outdoors for SUs engaging in exposure interventions and other types of therapy. These are important elements in supporting SUs’ progress toward community reintegration. Having organisational processes in place such as leave requirements are also unique to inpatient services and presented a novel barrier to outdoor working. These findings have not been highlighted in previous research and therefore add to the literature and need to be considered in clinical practice when working with this population.

### Limitations

Service users were interviewed by a psychologist who was not their regular psychologist in an attempt to minimise bias and demand characteristics. However, SRS were completed with SUs’ regular psychologists during therapy and therefore may have influenced the validity of SUs’ responses. Further, SRS scores must be interpreted with caution as the psychometric properties of the SRS were tested using community samples and therefore are not necessarily generalisable to the client group of this study. The need for further research to investigate the validity of the SRS with more specific clinical populations has been highlighted (Murphy et al., 2020).

The findings of this study have been collected solely from SUs who participated in outdoor therapy. This is likely to have resulted in the participants’ views of outdoor working intrinsically being more positive than those who declined outdoor sessions. The reasons for SUs opting out of outdoor therapy were not explored in this study, nor were the organisational factors that prevented participation (e.g., risk assessment processes and leave restrictions). Future research is warranted that explores these factors to gain a more multifaceted understanding of the facilitators and barriers within inpatient services.

### Implications for Practice

This study supports previous research suggesting person-centred therapy includes an openness toward alternative therapy environments (see “environmental safe uncertainty”; Cooley et al., 2021). Outdoor therapy offers the opportunity to connect with natural environments, which has been identified as important in supporting individuals to access a higher quality of life (Kellert and Wilson, 1994). Outdoor working is also in line with the core standards expected of inpatient mental health services, which cite SUs have the right to access “evidence-based interventions, which are appropriate to their bio-psychological needs” (The Royal College of Psychiatrists, 2019, p.8). Further, this mode of therapy increases the application of a holistic rehabilitation approach as outdoor working supports SUs with their emotional, physical and social wellbeing (Harper and Dodub, 2020). Finally, SUs have reported finding the everyday life on inpatient wards under stimulating, resulting in increased rumination and decreased wellbeing (Foye et al., 2020). Conversely, inpatient wards can at times be highly emotive environments which may increase SU stress. Outdoor sessions are therefore an opportunity to provide respite from the mundane of the everyday, as well as periods of higher stress.

## Conclusion

This feasibility study concluded that outdoor psychology sessions were a viable response to the COVID-19 pandemic within an NHS adult inpatient mental health rehabilitation service. Outdoor therapy was described as a beneficial experience for both SUs and psychologists, with SUs further reporting high satisfaction in therapeutic alliance and comfort outdoors. This provides evidence for the continuation of outdoor therapy as a treatment option within the service, provided effective risk assessment and management is conducted throughout the course of therapy.

## Data Availability Statement

The raw data supporting the conclusions of this article will be made available by the authors, without undue reservation.

## Ethics Statement

The studies involving human participants were reviewed and approved by Leicestershire Partnership NHS Trust WeImproveQ. The patients/participants provided their written or verbal informed consent to participate in this study.

## Author Contributions

KK, KF, and SC contributed to the research question generation and designing of the methodology and created the study documents including the bespoke interview schedules. KK and KF submitted the project for approval with the appropriate committee. KK, KF, and GJ identified service users who met the inclusion criteria to complete session rating scales and interviews. GJ and KK completed interviews and maintained a record of the completed session rating scales and diaries. GJ led the analysis which was reviewed by KK, KF, and SC. GJ wrote, revised and synthesised revisions of the manuscript from KK, KF, and SC. All authors contributed to the article and approved the submitted version.

## Conflict of Interest

The authors declare that the research was conducted in the absence of any commercial or financial relationships that could be construed as a potential conflict of interest.

## Publisher’s Note

All claims expressed in this article are solely those of the authors and do not necessarily represent those of their affiliated organizations, or those of the publisher, the editors and the reviewers. Any product that may be evaluated in this article, or claim that may be made by its manufacturer, is not guaranteed or endorsed by the publisher.

## Abbreviations

BPS: british psychological society
MDT: multi-disciplinary team
NHS: national health service
SRS: session rating scale
SU: service user.
